# 250 Objectively assessed sit-to-stand reserve is associated with difficulties in activities of daily living (ADL) and instrumental activities of daily living (iADL) among community-dwelling older adults

**DOI:** 10.1093/eurpub/ckae114.274

**Published:** 2024-09-26

**Authors:** Antti Löppönen

**Affiliations:** Faculty of Sport and Health Sciences and Gerontology Research Center, University of Jyväskylä, Finland

**Keywords:** accelerometer, relative, functional capacity

## Abstract

**Purpose:**

Physical reserve acts as a buffer against declines in physical function and can help maintain and restore it in acute situations that threaten physical function, such as illness. Previous studies have mainly focused on the difference between habitual and maximal walking speeds in the laboratory, neglecting the physical demands of free-living environment. Wearable sensors allow for a more comprehensive investigation of daily activities, including strength-demanding daily activities such as sit-to-stand (STS) transitions. This study investigates how STS reserve is associated with difficulties in activities of daily living (ADL) and instrumental activities of daily living (iADL) among community-dwelling older adults.

**Methods:**

The study involved 230 individuals aged 79 to 89 years old, whose capacity for STS transitions was measured using the instrumented STS test and whose free-living STS transitions were continuously monitored for 24 hours a day for 4 days using a tri-axial accelerometer (UKK RM42). Variables associated with STS reserve and self-reported ADL/iADL difficulties were analyzed using multinomial logistic regression.

**Results:**

Among the participants, 32.2% reported difficulties with one or two ADL/iADL functions, and 14.8% reported difficulties in three or more ADL/iADL functions. STS reserve was significantly associated with difficulties in one or two ADL/iADL functions (odds ratio [OR] = 0.83; 95% confidence interval [CI] = 0.70-0.97, per 10 deg/s increase) and three or more ADL/iADL functions (OR = 0.59; 95% CI = 0.47-0.74, per 10 deg/s increase). After adjusting for baseline age, sex, self-reported pain, cerebrovascular diseases, MMSE, balance test, and walking speed, STS reserve remained significantly associated with difficulties in three or more ADL/iADL functions (OR = 0.72; 95% CI = 0.54-0.97, per 10 deg/s increase).

**Conclusions:**

The findings suggest that reserve determined by strength-demanding STS transitions is associated with ADL/iADL difficulties, indicating that a strong reserve may enable unrestricted independent living.

**Support/Funding Source:**

This study was supported by the Academy of Finland, the Finnish Ministry of Education and Culture and an Advanced Grant from the European Research Council.

